# HPV Genotyping of Modified General Primer-Amplicons Is More Analytically Sensitive and Specific by Sequencing than by Hybridization

**DOI:** 10.1371/journal.pone.0169074

**Published:** 2017-01-03

**Authors:** Roger Meisal, Trine Ballestad Rounge, Irene Kraus Christiansen, Alexander Kirkeby Eieland, Merete Molton Worren, Tor Faksvaag Molden, Øyvind Kommedal, Eivind Hovig, Truls Michael Leegaard, Ole Herman Ambur

**Affiliations:** 1 Department of Microbiology and Infection Control, Akershus University Hospital, Lørenskog, Norway; 2 Department of Research, Cancer Registry of Norway, Oslo, Norway; 3 Bioinformatics Core Facility, Institute for Cancer Genetics and Informatics, The Norwegian Radium Hospital, Oslo University Hospital, Oslo, Norway; 4 Department of Vaccines, Norwegian Institute of Public Health, Oslo, Norway; 5 Department of Microbiology, Haukeland University Hospital, Bergen, Norway; 6 Department of Informatics, University of Oslo, Oslo, Norway; 7 Department of Tumor Biology, Institute of Cancer Research and Institute for Cancer Genetics and Informatics, The Norwegian Radium Hospital, Oslo University Hospital, Oslo, Norway; 8 Division of Medicine and Laboratory Sciences, University of Oslo, Oslo Norway; 9 Department of Life Sciences and Health, Oslo and Akershus University College of Applied Sciences, Oslo, Norway; Universidad de Chile, CHILE

## Abstract

Sensitive and specific genotyping of human papillomaviruses (HPVs) is important for population-based surveillance of carcinogenic HPV types and for monitoring vaccine effectiveness. Here we compare HPV genotyping by Next Generation Sequencing (NGS) to an established DNA hybridization method. In DNA isolated from urine, the overall analytical sensitivity of NGS was found to be 22% higher than that of hybridization. NGS was also found to be the most specific method and expanded the detection repertoire beyond the 37 types of the DNA hybridization assay. Furthermore, NGS provided an increased resolution by identifying genetic variants of individual HPV types. The same Modified General Primers (MGP)-amplicon was used in both methods. The NGS method is described in detail to facilitate implementation in the clinical microbiology laboratory and includes suggestions for new standards for detection and calling of types and variants with improved resolution.

## Introduction

Persistent infection with certain human papillomavirus (HPV) types is strongly associated with cervical cancer development [1]. HPV types associated with cancer development in humans all belong to the genus alpha-HPVs [2,3]. Based on the degree of association with malignancy, HPVs can be divided into high-risk (HR) and low-risk types. Commonly, 14 HPV types are regarded HR types (HPV16, 18, 31, 33, 35, 39, 45, 51, 52, 56, 58, 59, 66 and 68) and 12 types as low-risk types (HPV6, 11, 40, 42, 43, 44, 54, 61, 72, 81, and 89) [2,4]. Types 16 and 18 are the most prevalent HR types and account for 70% of HPV infections found in invasive cervical cancer [5]. Furthermore, with the exception of HPV51 (alpha-5), HPV56 and HPV66 (alpha-6), all HR types belong to the phylogenetically related clustered species groups, comprising HPV16 (alpha-9) and HPV18 (alpha-7), accounting for ~75% and ~15% of the global cervical cancer burden, respectively [5–8].

Identification of HR HPV types has been included in cervical cancer screening, preferentially as a triage method for women with abnormal cytology, in order to better identify women in need for further follow-up. In addition, genotyping of HPV is primarily important in epidemiological studies, e.g. for monitoring the impact of HPV vaccination. In Norway, HPV vaccination was introduced in 2009 for girls in the 7th grade, as a part of the childhood vaccination program. In order to monitor vaccine effectiveness and potential vaccine-related changes in the HPV-type epidemiology, genotyping of HPV in urine samples from vaccinated and non-vaccinated girls and young women is undertaken [9].

Individual HPV types can be detected using DNA hybridization and Luminex^®^ technology in a high-throughput manner [10]. A protocol for the genotyping of 37 HPV types using hybridization of the modified general primer (MGP)-amplicon to a defined set of HPV specific probes by Luminex^®^ technology has been developed at the WHO-HPV LabNet Global Reference Laboratory in Malmö, Sweden, and was implemented at the Norwegian HPV Reference Laboratory in 2010 to monitor HPV vaccine effectiveness. An overview of HPV types detected by Luminex^®^ is presented in Table 1.

**Table 1:** Risk classification of HPV-types.

Classification	HPV types
Group 1 Carcinogenic (high-risk)	16, 18, 31, 33, 35, 39, 45, 51, 52, 56, 58, 59
Group 2A Probably carcinogenic (high-risk)	68
Group 2B Possibly carcinogenic (intermediate risk)	26, 53, 66 ^a^, 67, 70, 73, 82
Group 2B Unknown risk ^b^	30, (34), 69, (85), 86, (97)
Group 3 Low-risk	6, 11, 40, 42, 43, (44), 54, 61, (71), (72), 81, 89, 90 ^c^
Not included in IARC 2012 classification (unknown risk/low- risk)	HPV 74, 83, 87, 91

HPV types included in the Luminex-assay, combined and grouped according to IARC 2012 risk classification. HPV types in brackets are not included in the Luminex-assay.

^a^Classified as carcinogenic (high-risk) in IARC 2007

^b^Classified in this group based on their phylogenic analogy to HPV types with sufficient or limited evidence of carcinogenicity.

^c^Not specified in IARC 2012.

The Ion Torrent™ (Life Technologies) Personal Genome Machine (PGM) is a bench-top apparatus that sequences DNA by measuring released protons in DNA polymerase nucleotide incorporation events. The cost of NGS sequencing has dropped considerably the past few years [11], making it competitive with traditional hybridization approaches.

Several clinical and experimental HPV-typing assays are available [12]. Many of these assays, including the Luminex assay, are based on consensus primers to amplify a conserved region of the *L1* gene in the HPV genome, followed by DNA hybridization to HPV-specific probes for type specific detection. The MGP PCR-system consists of five forward and five reverse consensus primers that, depending on HPV type, amplifies 158–168 nt of the *L1* gene, and has been shown suitable and sensitive for genotyping of HR HPV types, including co-infections with multiple HPV types [13,14]. The MGP primers are well suited for epidemiological studies and vaccine surveillance, since they capture all 14 HR-HPV types including the 4–9 different vaccine types, the 12 low risk types and overall more HPV genotypes than amplicons obtained from other primer sets [13]. The MGP primers are based the GP5+/6+ primers [15–17] and were further developed by the WHO HPV LabNet Global Reference Laboratory [13]. Similar studies reported at the time of planning this study, typically combined the HPV consensus primers MY09/11,PGMY or a modified PGMY, which amplify a 450 nt fragment, in combination with 454 sequencing technology [18–20]. A recent study utilized the Ion Torrent platform in combination with the HPV broad-spectrum general primers (BSGP5+/6+) on formalin-fixed, paraffin-embedded cancer specimens, showing high sensitivity and specificity [21]. The BSGP5+/6+ primer system was designed by Schmitt et al. [22] and consists of a pool of 3 reverse primers and 9 forward primers targeting the *L1* gene. Other recent studies using NGS for HPV genotyping have designed and utilized new multiple degenerate primers for amplification targets in L1 or even targets in the E6/E7 region of the HPV genome [23,24].

The MGP primer-set was chosen in order to get a direct comparison between NGS and the established Luminex assay, with the aim to directly compare these technologies on urine samples, in which a higher frequency of multiple infections is expected. Urine samples were chosen since these are investigated in the HPV vaccine surveillance program in Norway [9].

## Materials and Methods

### Sample material

Residual urine (1 ml) was collected from 104 samples submitted for routine diagnostics of *Chlamydia trachomatis*. Their use in the comparative quality assessment study was approved by the Data Protection Official for Research at Akershus University Hospital. The inclusion criteria were as follows: fresh sample (<4 days from sampling), from women in the age range 18–35 years. Samples were anonymized upon selection and kept at -80°C until DNA extraction. A complete flow chart of sample processing for hybridization and NGS assays are given in S2 Fig.

As positive controls, the 1^st^ WHO International Standards for HPV16 DNA and HPV18 DNA (National Institute for Biological Standards and Control, code: 06/202 and 06/206, respectively) were used, as well as DNA from the cancer cell lines CaSki (HPV16), HeLa (HPV18) and SiHa (HPV16). As background DNA in controls and for the generation of a standard curve, human genomic DNA (Roche Life Science, Indianapolis, Indiana, USA) was used.

### Nucleic acid extraction and quality control

Nucleic acid extraction was performed using an automated system, NucliSENS easyMAG (BioMérieux INC, France), and offboard lysis. Nucleic acids were eluted in 70 μl elution buffer and stored at -80°C prior to HPV analyses. To evaluate sample adequacy, the human β-globin gene was amplified using a TaqMan assay with 0.2 μM of the modified PCO3 14-39F and PCO4 123-103R primers, together with 0.04 μM of the β-globin 55–85 probe for detection on a qPCR system, Stratagene Mx3005P (Agilent Technologies, Santa Clara, CA, USA), as previously described [25,26]. Sample input was 5 μl, to a total reaction volume of 25 μl. For quantification, a tenfold dilution series of β-globin (ranging from 100,000 copies to 1 copy per reaction) was included for standard curve generation. Reactions with water instead of template were included as negative controls.

### HPV amplicon generation

All samples were analyzed for the presence of HPV by PCR, using the MGP primer set as previously described by Söderlund-Strand *et al*. [13]. The five forward and the five reverse MGP primers amplify 158–168 nt of the *L1* gene depending on the HPV type. In the MGP PCR protocol for Luminex^®^, the AmpliTaq Gold polymerase (Life technologies/Applied Biosystems, CA, USA) was used. A modified MGP PCR protocol using the proofreading Phusion Hot Start II High-fidelity DNA polymerase (Thermo Fisher Scientific, MA, USA) was established for NGS. Here, the modified MGP PCR mastermix consisted of: 1 × Phusion HF buffer, 0.2 mM dNTPs, 1 μM primer mix, 0.2 U Phusion DNA polymerase. Sample input was 5 μl to a total reaction volume of 20 μl with a final concentration of 0.1 μM of each primer. The modified MGP PCR protocol was run with the following cycling conditions: 98°C 30 s, 5 cycles 98°C 5 s, 42°C 5 s and 72°C 5 s, followed by 45 cycles 98°C 5 s, 64°C 5 s, 72°C 5 s prior to cooling to 4°C. All samples were stored at -20°C until further processing and analysis. To evaluate the outcome of each PCR, 5 μl of all PCR products were analyzed on a 4% E-gel EX (Life technologies/Invitrogen, CA, USA).

### Luminex^®^ HPV genotyping of 37 HPV types

HPV type-specific DNA probes covalently linked to fluorescence-labeled polystyrene beads (MAPx) were hybridized to HPV amplicons labeled with a spectrally distinct fluorophore and these hybrids were detected by two lasers that excite the two fluorophores in Luminex^®^ as described by Schmitt et al., [10]. In the Luminex^®^ assay, 42 probes were used for the specific detection of 37 HPV types. Two different probes were used for the detection of each HPV 35, 58 and 68 representing different genetic variants. Non-typeable additional types were amplified with two universal primers. These probes included the 24 described in Schmitt et al., 2006 [10] and Söderlund-Strand et al., 2009 [13] with some modifications, in addition to 18 new probes obtained from the WHO HPV LabNet Global Reference Laboratory in Malmö, Sweden (S1 Table). The Luminex^®^ method was performed as described previously [10,13]. The performance of the Luminex^®^ assay at The Norwegian HPV reference laboratory to detect HPV 6, 11, 16, 18, 31, 33, 35, 39, 45, 51, 52, 56, 58, 59, 66, 68a and 68b has been monitored annually using the external quality assessment program EQUALIS with proficient results [27,28].

### Library construction, quality control and NGS

Libraries for NGS were generated using protocols from Life technologies (Life technologies, Carlsbad, CA, USA) provided through the “Ion community” web-portal. The protocol “*Preparing Short Amplicon (<350 bp) Libraries Using the Ion Plus fragment Library kit*” (MAN0006846, Revision 3.0), was used with the following modifications: To 13 μl of PCR product, nuclease free water was added to a total of 50 μl before cleanup using Agencourt AMPure XP (Beckman Coulter, CA, USA). Samples were quantified using the Qubit 2.0 fluorometer and the dsDNA HS Assay kit (Invitrogen) before end-repair. Adapters were ligated according to the manufacturer’s instructions (MAN0006846, Revision 3.0) followed by cleanup using 1.6 × AMPure XP (Beckman Coulter, CA, USA). Sample libraries were diluted 1:10 and 1 μl was used for quantification and quality control on the Agilent 2100 Bioanalyzer (Agilent Technologies, CA, USA), using the High Sensitivity DNA kit prior to pooling each batch of 32 barcoded sample libraries at about 5000 pM per sample library. The pooled libraries were adjusted to 100 μl using Nuclease free water for two final rounds of 1.4 × AMPure XP cleanup with elution for the first cleanup in 30 μl, and then in 15 μl nuclease free water for the second cleanup. The library dilution factor was determined using 1 μl of the purified library samples on the Agilent 2100 Bioanalyzer as described above. To reduce the amount of polyclonal reads, the input library concentration for sequencing template generation was reduced from 26 pM to 15 pM. Generation of sequencing libraries was performed as described in the manual “*Ion PGM*^*TM*^ *Template OT2 200 Kit*” (MAN0007220, Revision 3.0). Sequencing of the libraries was performed on the Ion Torrent™ PGM using the manual “*Ion PGM*^*TM*^ *Sequencing 200 Kit v2*” (MAN0007273, Revision 1.0) and the Ion 314^TM^ Chips. All libraries were loaded to the chips according to the manufacturer’s instructions as specified in “*Simplified Ion PGM*^*TM*^ *Chip Loading with the Ion PGM*^*TM*^ *Weighted Chip Bucket*” (MAN0007517, Revision 1.0).

### NGS data analysis

Data were obtained from the Ion Torrent™ sequencer as de-multiplexed fastq files. Computer analysis was performed using a custom data analysis pipeline that first removed adapters, short reads (<50 bases) and low quality bases (<13) using Cutadapt v1.10 [29]. The remaining sequence reads were then aligned to the genome using Bowtie2 v2.2.9 [30]. Bowtie2 searched for alignments using all of the read characters in an “end-to-end” mode. At most three distinct, valid alignments for each read were searched for and only the best alignment was reported. Zero mismatches were allowed in the 22-nucleotide long seed alignment. The interval between seeds were set by the function f(x) = 1 + 2.5 * sqrt(x), where x is the read length. These settings were equal to the preset “sensitive” setting in end-to-end mode. All preset settings in Bowtie2 were tested on a mock dataset to optimize the alignments. At the time of analysis, the alignment reference consisted of 175 verified HPV-type reference HPV genomes listed at the international HPV reference center (www.hpvcenter.se/html/refclones.html). Variants of individual HPV types differing from the HPV type reference were not included in the alignment reference. The HPV genome sequences were downloaded from the NCBI Genbank database. A count of sequence reads that mapped to each reference genome were calculated by FeatureCount from the Subread package v1.4.6 [31]. Alignments and counting were run on 12 parallel search threads. To identify as many true positives as possible and at the same time keep false positives at a minimum, a treshold of 20 reads aligned to a reference was set for positive HPV genotype assignment [32]. The NGS pipeline is summarized in S4 Table. NGS cut-off depth values of 5, 10, 20, 50 and 100 were investigated in regard to changes in methods agreement.

### Sensitivity testing

Both hybridization (Luminex®) and NGS (Ion Torrent™) technologies were used to analyze all samples and controls. Analytical sensitivity was investigated by analyzing dilution series of the international standards for HPV16 and HPV18. Analytical sensitivity was also investigated for HPV16 in the presence of varying concentrations of background human genomic DNA (β-globin). A constant of 500 copies HPV16 DNA was tested in the presence of 1000 copies β-globin to yield more than 95% HPV-sequences by NGS, dropping to about 50% HPV-sequences when mixed with 10,000 copies β-globin. Most sequencing reads that were not HPV and hence background noise, mapped to human genomic DNA. Saturation by human DNA was monitored up to 100,000 copies β-globin, where HPV16 was no longer detected. HPV16 was detected in the positive control cell-lines CaSki and SiHa and HPV18 in the HeLa cell-line. Confirmation of analysis results; sensitivity and specificity calculations. All single identifications made with just one of the two methods were confirmed with a third method using either real time PCR with HPV type-specific primers (HPV6, 16, 18, 31, 33, 35, 39, 42, 43, 45, 51, 52, 56, 58, 66, 68, 90) and SYBR^®^ Select Master Mix (Thermo Fischer Scientific) or by sequencing of a 450 bp PGMY amplicon [18,19] on a MiSeq (Illumina) platform (HPV30, 32, 40, 53, 54, 62, 67, 73, 74, 82, 87, 89, 91 and 114). Type-specific HPV primers were designed using Primer3 [33] for HPV types: 68 (forward: 5’-tcacgagcaattagragatt-3’, reverse: 5’-gtctggctagtagtwgatgt-3’, T_A_ = 54.2°C) and 90 (forward: 5’-tgttttgcggaacagcatta-3’, reverse: 5’-cttagtttcccggctgcttc-3’, T_A_ = 51.9°C). All other primers and their PCR conditions are described elsewhere, for HPV types: 16, 18, 33 and 35 [34]; 42, 43 and 66 [35]; 6, 39, 51 and 56 [36]; 31, 45, 52 and 58 [37]. The overall sensitivity or true positive rate for each method was calculated by dividing the total number of true positives by the total sum of true positives and false negatives. The false discovery rate was calculated by dividing the number of false positives by the sum of true and false positives. Each HPV type specific agreement (%) was calculated by dividing the sum of correlating positive and negative results by the total number of samples (n = 103). The overall correlation between the two methods was measured by calculating the correlation coefficient from the distributions of true corresponding positive and negative identifications using the CORREL function in Excel. The coefficient therefore reflects all deviating results not limited to true confirmed identifications. A perfect negative correlation with a correlation coefficient of -1 is expected if the two methods perform identically.

## Results

Of the 104 clinical samples, 103 were positive for human DNA by *β-globin* PCR and hence found appropriate for further analysis. The frequencies of the total available reads (HPV and others) in all samples by NGS (minimum: 97, maximum: 28658, median: 11672) were very similarly distributed as those in the HPV positive (minimum: 116, maximum: 28658, median: 11675) and HPV negative samples (minimum: 97, maximum: 21466, median: 11058) (S2 Table). In contrast, the frequencies of the HPV aligned reads in HPV positive samples (minimum: 30; maximum:19166; median: 6335) differed considerably from those of HPV negative samples (maximum: 6) (S2 Table and S1 Fig). A 20 read depth cut-off was set based on the minimum 18 read-depth cut-off recommendation in [32] and evaluated against the steady decrease in “NGS alone”- and “Both methods”-detections and increase in “Hyb. alone”-detections with increasing cutoff as shown in Table 2.

**Table 2:** The influence of NGS cut-off values on HPV detections made by NGS and hybridization. (The 20 read depth cut-off in **bold**).

NGS cut-off	Both methods	NGS alone	Hyb. alone	Both negative
**5**	185	120	4	3435
**10**	181	104	8	3451
**20**	**178**	**84**	**11**	**3471**
**50**	170	52	19	3503
**100**	159	38	30	3517

**Fig 1:**
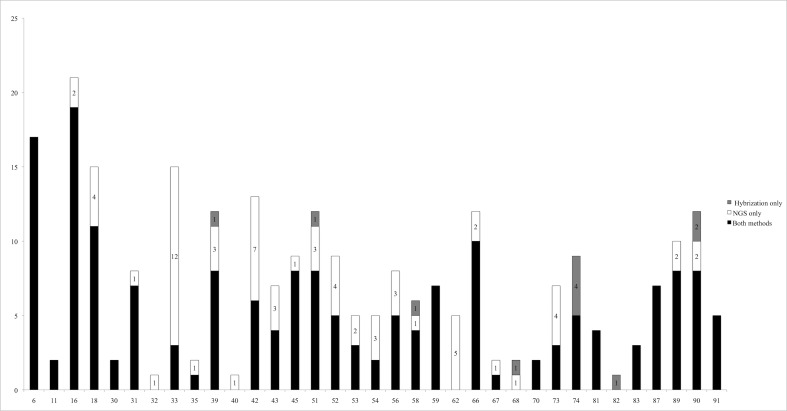
HPV type-specific detections by hybridization and NGS. The number of HPV detections in 85 urine samples is given on the Y-axis and the individual HPV types are given on the X-axis. Black bars show detections made by both methods; grey bar sections show detections made by hybridization only and white bar sections show detections made by NGS only.

Positive control samples consisting of HPV type reference plasmids (HPV16 and 18) and cell lines showed 100% agreement in regard to the detection limits for the two methods and with the 20 read depth NGS cut-off. HPV16 was detected down to the level of 500 plasmid copies and HPV18 to a level of 50 plasmid copies. In total, 256 confirmed HPV genotype detections comprising 35 HPV types were made in 85 of the 103 urine samples (Fig 1 and S3 Table). NGS made 247 HPV type detections whereas hybridization made 189. 178 identifications were the same by both methods. The correlation coefficient between positive and negative identifications made by both methods was -0,66. The overall sensitivity was 73,8% by hybridization and 95,7% by NGS. Taken together, NGS therefore displayed 22% higher sensitivity than the hybridization method. In addition, 26 detections were made that were not confirmed by other methods, of which 15 were made by NGS and 11 were made by hybridization. On this basis the false discovery rate was 5,5% for hybridization and 6,1% for NGS. The 11 unconfirmed calls made by hybridization were for HPV types 6, 33, 67, 68, 82, 90 and 91 (S3 Table). The 15 unconfirmed calls made by NGS were for HPV16, 30, 31, 32, 33, 35, 45, 56, 74, 87 and 114 (S3 Table). The unconfirmed detections are not included in figures or tables unless otherwise stated. The majority of samples contained more than one HPV type as shown in Table 3.

**Table 3:** Multiple HPV types detected in 103 urine samples.

Total HPV types/sample	Number of samples
0	18
1	19
2	21
3	14
4	9
≥5	22

HPV was exclusively detected by either NGS or hybridization in three *urine samples*: a detection of HPV39 by hybridization, a detection of HPV16 by NGS and detections of HPV18, 33, 52 in one sample also by NGS (S3 Table). Detections of HPV32 (1 sample), HPV40 (1 sample) and HPV62 (5 samples) were exclusive to NGS and a single detection of HPV82 was exclusive to hybridization (Fig 1). Identical performances without single-method or unconfirmed identifications were found for HPV11, 59, 70, 81 and 83. The poorest agreement between the methods was 73.8% observed for HPV33. HPV33 was detected by both methods in three samples, in addition to twelve detections only by NGS. The second lowest correlation between the methods was 86.4% observed for HPV42. HPV42 was detected by both methods in six samples; seven additional detections were made only by NGS. Hybridization made confirmed identifications of HPV39, 51, 58, 68, 74, 82 and 90 that were not made by NGS, and NGS made confirmed identifications of HPV16, 18, 31, 33, 35, 39, 40, 42, 43, 45, 51, 52, 53, 54, 56, 58, 62, 66, 67, 68, 73, 89, and 90 that were not made by hybridization. It is notable that NGS detects more of the clinically important vaccine type HPV18 to which the two methods display 92.2% agreement (S3 Table). NGS also detects more HPV16, the most common and clinically important type also included in HPV vaccines. The agreement between the two methods for detecting HPV16 was 95.1% (S3 Table). Hybridization makes four more detections of HPV74 than NGS (Fig 1).

Genetic *variants* of HPV16, 30, 32, 43, 51, 52, 53, 73, 74, 87 and 90 were identified by NGS. Two detections of HPV16 made by NGS displayed genetic polymorphisms that were located in the probe region used for detection in the hybridization assay. All nucleotide polymorphisms (NPs) identified in HPV43, 51, 52, 53, 73 and 90 were also located in the probe regions of their respective type-specific probes. For five out of the nine HPV74 identifications made by NGS, the MGP-amplicon DNA sequence revealed perfect matches with hitherto unclassified HPV-types (EU911664, EU911625 and HQ834629) that were identical to HPV74 in the much shorter probe-binding region. Similar observations were made for individual detections HPV30, 32 and 87.

## Discussion

Testing for the presence of HPV in urine samples is a suitable, reliable and noninvasive way to obtain epidemiological data for e.g. vaccine surveillance [38]. The majority of the included urine samples were HPV positive (85/103). A higher than average HPV prevalence was expected from studying samples collected for diagnostics of *Chlamydia trachomatis*, which transmit along the same route as HPV, i.e. sexually. The sample set therefore likely represents a population with higher risk of HPV infection(s) than expected in the average population. The methodological differences in analytical sensitivity described here may therefore particularly apply to urine with high prevalence and co-infections of HPV. The results presented here have no clinical outcome to correlate to and are therefore limited to analytical correlation with other test methods. It is therefore not possible to interpret the clinical significance of correlating and non-correlating results. Analytically NGS was here shown the most sensitive method. It may be that the high number of positives exclusively detected by NGS are of less clinical importance than correlating results or less sensitive methods. The sensitivity of clinical HPV tests used in cervical screening programs depend on parameters such as the viral load [39]. As addressed by Vorsters et al., (2016) [40] investigations of the potential clinical value of urine viral load are warranted and the methods presented here may prove helpful in such future investigations.

Amplification of DNA by PCR always introduces amplification biases, and it is known that the observed prevalence of HPV analyzed from the same set of samples can vary between different assays [14,41]. Such biases become accentuated when the PCR reaction contain both multiple HPV types and primers [42]. To keep the amplification biases at a minimum, we used the same MGP amplification primers for both NGS and hybridization. Multiple HPV types were detected in the majority of the samples and may partly account for discrepant results. One sample contained as much as 11 different HPV types of which 4 were detected by NGS alone (S3 Table). In complex samples with several HPV types one may suspect that both cross-hybridizations and internal competition for signaling chemistry and probes can mute signals in the Luminex-assay to undetectable levels. HPV typing by NGS is different in that it is not dependent on probes for detection that may cause cross-hybridizations. At least three previous HPV-genotyping assays using NGS have found NGS to be both sensitive and accurate: Arroyo et al. and Militello et al. both used the PGMY set of primers for the amplification of a 450 bp fragment [18,19], while Yi et al. [43] used a series of unique primers for the amplification of a 150 bp fragment of the *L1* gene, which is similar in length to the fragments produced by the MGP primers used here.

The relative amount of human background DNA to HPV DNA was also shown to affect the ability to detect HPV, probably due to unspecific DNA amplification. This assumption is supported by the NGS data from the control samples displaying the relative amount of co-amplified human background DNA sequences (S1 Fig).

### Correlation and discordance in HPV typing between NGS and hybridization

A strong overall correlation between the two methods was documented when the statistical analysis included both the true corresponding positive and negative identifications. The 2010 global proficiency study of human papillomavirus genotyping in vaccinology [27] also measured specificity of HPV typing as absence of detection of a specific HPV-type when also other HPV types were present. It is notable however, that considerable variation in HPV type specificity measured as agreement between the methods. Particularly, low agreement was observed for HPV types 33 and 42, in addition to those HPV types detected by either method alone (HPV32, 62 and 82). Probes matching HPV32 and 62 were not part of the Luminex panel. Expanding the panel to include more HPV types would require rigorous testing of the sensitivity of *all* HPV types in the assay, due to the potential for cross-hybridization with a new mixture of probes.

The hybridization method was considerably less sensitive than NGS in detecting HPV33, with only three detections vs. fifteen (Fig 1). Cervical HPV33 prevalence among European women (normal population) has been reported to be one of the five most common types, alongside HPV16, 31, 18, 39, and 66 [44–46], whereas hybridization in this study reports HPV33 as not among the fifteen most common types. NGS findings are therefore more in harmony with the European prevalence when reporting HPV33 as the third most common type. In addition to being among the most prevalent HPV types in high-grade cervical intraepithelial neoplasia and cervical cancer in Europe [47], HPV33 is also reported to have a clinical high positive predictive value for high-grade lesions comparable with that of HPV16 [48]. Since the beginning of the Norwegian vaccine surveillance project in 2009, a new HPV33 probe has been designed at the WHO HPV LabNet Global Reference Laboratory (personal communication), suggesting that the older HPV33 probe used in this study may be further optimized.

More than 50% of the thirteen calls made for HPV42 were discordant (Fig 1). NGS detects HPV42 in 13 samples and hybridization in six. All six detections made by hybridization were also detected by NGS. Although there were no detected mismatches, redesign of probe(s) for HPV42 and/or minor adjustments in the cut-off could potentially improve the specificity and sensitivity for HPV42 detection by the hybridization method.

Of the twenty-two detections of the clinically important HPV16, two were made exclusively by NGS. Failed detection by hybridization is likely to have resulted from a mismatch between the hybridization probe and the HPV16 variant uniquely present in these samples (marked with X in S3 Table). Also the presence of HPV66 in one of these samples may have influenced hybridization, due to cross-reactivity and correction thereof in the Luminex- algorithm. The identification of HPV16 *variants* by NGS may also prove important in clinical settings, since great variability in precancer/cancer risk has been found in different lineages of the same type [49]. Also, since HPV vaccines are targeted against the *L1* gene, their efficacy against individual variants could potentially vary.

HPV18, also of great clinical importance, was detected exclusively by NGS in four out of 15 calls. Two of these could potentially have been missed by hybridization due to cross-reactivity with HPV45 present in these samples. HPV45 is very similar at the sequence level and the HPV type most closely related to HPV18 [50]. Weak HPV18 signals below cut-off were observed in the two latter samples and a minor revision of cut-off could potentially have made positive calls. However, such an alteration would also require adjustment of cross-hybridization correction factors in the Luminex-algorithm influencing the cut-off of other HPV types e.g. HPV45. All four samples with discordant HPV18 results contained multiple HPV types that will compete in the PCR to affect which types that reaches a threshold where a positive call is made. Hybridization was particularly more sensitive in detecting HPV74 with four more detections than NGS. Three of these displayed read counts (5, 5 and 14) below the 20 read depth cut-off by NGS (S4 Table). A type specific cut-off at 5 reads for HPV74 would have captured these and one additional unconfirmed sample. Together with HPV40 and 68, HPV74 displays the lowest average number of reads per HPV type (S4 Table) and might reflect relatively poorer amplification of these types by the MGP primers. Since the MGP and other multiplexing HPV amplicons amplify different HPV types with different efficiency as described in [14], type specific cut-offs should be considered for individual amplicons in future studies. Potentially different cut-offs may also be applied to different types of samples such as tissue, liquid based cytology, saliva and urine as the viral load and HPV type multiplicity is expected to vary.

Four or less discordant calls were made by both methods for a range of different HPV types: HPV32, 35, 39, 40, 43, 45, 51, 52, 53, 54, 56, 58, 66, 67, 68, 73, 74, 82, 87, 89 and 90. Hybridization made identifications of HPV39, 51, 58, 68, 74, 82 and 90 that were not made by NGS and NGS made identifications of HPV16, 18, 30, 33, 35, 39, 40, 42, 43, 45, 51, 52, 53, 54, 56, 58, 62, 66, 69, 73, 89, and 90 that were not made by hybridization. In addition to PCR stochasticity, particularly in the first cycles of competitive amplification of individual types, some discordant results may be associated with probe mismatches to genetic variants (HPV16, 30, 32, 43, 51, 52, 53, 73, 74, 87 and 90). Yet, others may be caused by cross-hybridization events. For example HPV82 detected by hybridization has been shown to cross-hybridize with HPV18 and 51 (personal communication WHO HPV LabNet Global Reference Laboratory). HPV51 was detected in the samples calling HPV82 by hybridization. Although the HPV52 and HPV82 probes are different (S1 Table) cross hybridization may happen since 19 nucleotides out of the 22 nucleotides of the HPV82 probe matches perfectly to a continuous stretch in HPV51. Hybridization made single detections of HPV39, 58 and two detections of HPV90 where NGS produced reads (5, 15, 16 and 9, respectively) below the read depth cut-off and points to the potential for HPV type specific cut-offs discussed above. However, the HPV39 call may have been caused by well-to-well contamination during DNA extraction since the neighboring sample produced >500 times more HPV39 reads (2556) (S2 Table).

Generally, the NGS approach was considerably more sensitive than the hybridization method (22%). The 20 read depth cut-off used in NGS may be, at least for certain HPV types, relatively lower than individual type specific cut-offs in the hybridization algorithm. Setting the NGS cut-off to 20 read depth was based on recommendations from others [32] and from considering the “cost of loosing” and “price of gaining” correlating detections. At the 20 read cut-off 69 of the 84 “NGS alone” detections (= 82%) were confirmed by other methods, the number of “hyb. alone” detections were limited to 11 and 178 detections (69.5%) of all 256 were the same by both methods. Reducing the cut-off by half to 10 reads was considered too costly since the number of detections made by both methods only increased by 3 to just 181 (70.7%) whereas the number of NGS alone detections increased by 20 detections.

### Unconfirmed results

It is difficult to completely disregard the 15 unconfirmed NGS results, since the sequencing reads actually are present in these samples and the output on average was considerably above the 20 read depth cut-off (S2 and S3 Tables). Possibly were the validating methods (type specific real time PCR and PGMY amplicon sequencing) in some of these instances insufficiently sensitive relative to the MGP amplicon. This may particularly apply to the detection of HPV114 with 40 reads in sample 87 (S2 and S3 Tables) where validating methods remains to be documented or further developed. Also, cross-contamination between wells or tubes during extraction cannot fully be excluded in certain cases and is always a risk in assays based on very sensitive molecular methods using amplification of nucleic acids. A single unconfirmed NGS call for HPV16 may have been caused by such contamination, since the number of HPV16 reads was low (168) compared to the neighboring sample (6150) (S2 and S3 Tables). The same phenomenon may have applied for individual unconfirmed hybridization calls for HPV6, 33, 87 and 91 (S2 and S3 Tables).

Hybridization made three unconfirmed calls for HPV68 which are probably caused by cross-hybridizations. A new probe for the detection of HPV68 has been designed at the WHO HPV LabNet Global Reference Laboratory (personal communication) since the start of the surveillance the Norwegian childhood vaccination program, suggesting that the HPV68 probe used in this study also has potential for optimization.

## Conclusions

A direct comparison between hybridization and NGS for HPV genotyping of urine samples was made that used the same amplicon. Although an overall good correlation between the two methods was shown, this applied only when both negative and positive results were regarded together. Considerable variation between the two methods was documented in both the sensitivity for making confirmed detections of individual HPV types and in the specificity for making correct species designations. NGS was found to be the most sensitive method in making 22% more confirmed detections than hybridization. NGS was also the most specific method in being independent of probes and therefore able to differentiate closely related HPV types from each other and to identify genetic variants. The clinical impact of these findings remains to be explored.

## Supporting Information

S1 Table Probes of the Luminex^®^-assay. (PDF)

S2 Table Count Table NGS and Read Count Summary. (XLSX)

S3 Table HPV type detections in each urine sample detected with NGS and/or hybridization. (PDF)

S4 Table Schematic overview of the NGS bioinformatic pipeline and data analysis. (PDF)

S1 Fig Read count frequency plot of total count of HPV-aligned reads vs. total number of available reads. (TIF)

S2 Fig Flow chart of sample processing for hybridization and NGS assays. (PDF)
