# Supplementary material for: HPV Genotyping of Modified General Primer-Amplicons Is More Analytically Sensitive and Specific by Sequencing than by Hybridization
Source: PLoS One. 2017 Jan 3;12(1):e0169074. doi: 10.1371/journal.pone.0169074 (PMC5207713; doi:10.1371/journal.pone.0169074)
Supplement: S1 Table — (PDF) [file pone.0169074.s001.pdf]

S1 Table: Probes of the Luminex®-assay

Probe ID	Sequence
HPV 6	5’amino-modified C12- TCC GTA ACT ACA TCT TCC A 3’
HPV 11	5’amino-modified C12- TCT GTG TCT AAA TCT GCT AC 3’
HPV 16	5’amino-modified C12- TAC CTA CGA CAT GGG GAG 3’
HPV 18	5’amino-modified C12- TGC TTC TAC ACA GTC TCC T 3’
HPV 26	5’amino-modified C12- GTA CAT TAT CTG CAG CAT C 3’
HPV 30	5’amino-modified C12- TCT GCA ACC ACA CAA ACG TT 3’
HPV 31	5’amino-modified C12- GCA ATT GCA AAC AGT GAT AC 3’
HPV 33	5’amino-modified C12- TGC ACA CAA GTA ACT AGT GA 3’
HPV 35	5’amino-modified C12- CTG CTG TGT CTT CTA GTG A 3’
HPV 35 pos 6624:A	5’amino-modified C12- CTG CTG TGT CTA CTA GTG A 3’
HPV 39	5’amino-modified C12- TAC ATT ATC TAC CTC TAT AGA 3’
HPV 40	5’amino-modified C12- AGT CCC CCA CAC CAA CC 3’
HPV 42	5’amino-modified C12- GCC ACT GCA ACA TCT GGT G 3’
HPV 43	5’amino-modified C12- TCT ACT GAC CCT ACT GTG 3’
HPV 45	5’amino-modified C12- TAA TTT AAC ATT ATG TGC CTC 3’
HPV 51	5’amino-modified C12- TGC TGC GGT TTC CCC AA 3’
HPV 52	5’amino-modified C12- GAA TAC CTT CGT CAT GGC 3’
HPV 53	5’amino-modified C12- TGT CTA CAT ATA ATT CAA AGC 3’
HPV 54	5’amino-modified C12- CAC GCA GGA TAG CTT TAA T 3’
HPV 56	5’amino-modified C12- GAT GCA CGA AAA ATT AAT CAG 3’
HPV 58	5’amino-modified C12- TAT GCA CTG AAG TAA CTA AG 3’
HPV 58 6688A	5’amino-modified C12- TAT GCA CTG AAG TAA ATA AG 3’
HPV 59	5’amino-modifierad C12-AGA ATA TGC CAG ACA TGT G 3’
HPV 61	5’amino-modified C12- CCC TGT ATC TGA ATA TAA AGC 3’
HPV 66	5’amino-modified C12- CGT GAA ATC AAT CAA TAC CTT C 3’
HPV 67	5’amino-modified C12- CTA CAT ACA AAA ATG AAA AC 3’
HPV 68 (Orth)	5’amino-modified C12- GCT GTG TAT GAT TCT AAT AAA T 3’
HPV 68 (ME 180)	5’amino-modified C12-CTG AAT CAG CTG TAC CAA A 3’
HPV 69	5’amino-modified C12- CAT CTG CCA CTT TTA AAC C 3’
HPV 70	5’amino-modified C12- TTT ACA TTG TCT GCC TGC A 3’
HPV 73	5’amino-modified C12- GTA TGC CAA CTC WAA TTT TAA 3’
HPV 74	5’amino-modified C12 –CAG ACT ACA AAC AAT ACA TC 3’
HPV 81	5’amino-modified C12- GCT ACA TCT GCT GCT GC 3’
HPV 82	5’amino-modified C12- ACT CCA RCA AAC TTT AAG CAG T 3’
HPV 83	5’amino-modified C12- TGC TGC TAC ACA GGC TAA 3’
HPV 86	5’amino-modified C12- ATT AGT GCC GCT ACC CAG AA 3’
HPV 87	5’amino-modified C12- CCA CTG AAT ATG ACC CCA 3’
HPV 89	5’amino-modified C12- AAT ACA GTT CTA CAC GCT 3’
HPV 90	5’amino-modified C12- CAC ATA CAA GGC TTC CAA TT 3’
HPV 91	5’amino-modified C12- TAC CTA CTA CAT ATG ACA AC 3’
Universal 1	5’amino-modified C12- GiC ATG iiG ARG AAT ATG A 3’
