# Supplementary material for: HPV Genotyping of Modified General Primer-Amplicons Is More Analytically Sensitive and Specific by Sequencing than by Hybridization
Source: PLoS One. 2017 Jan 3;12(1):e0169074. doi: 10.1371/journal.pone.0169074 (PMC5207713; doi:10.1371/journal.pone.0169074)
Supplement: S3 Table — (PDF) [file pone.0169074.s003.pdf]

S3 Table: HPV type detections in each urine sample detected with NGS and/or hybridization

Urine sample number	HPV-types detected																																				Sum validated genotypes Luminex	Sum validated genotypes NGS
	6	11	16	18	30	31	32	33	35	39	40	42	43	45	51	52	53	54	56	58	59	62	66	67	68	70	73	74	81	82	83	87	89	90	91	114		
1								B															A										A				2	3
2																																B					0	0
3	A							B						A																			A				3	3
4			A	A																																	2	2
5			A	A											B		B				A						B										3	6
7																	B, X		A									A				A					3	4
8					B			C							C																						1	0
9	A					B										A							A												C		3	3
10	A		A					B					B, X																					B, X	A		3	6
12			A		B	A		B																A													3	4
13			B, X																																		0	1
14												A	B, X																			A	A				3	4
15				B										A				B				B												A			2	5
16				B				B								B																					0	3
18	A																																				1	1
19										A																		A									2	2
20																							A														1	1
21						A													A									C					A				4	3
22												A																							A		2	2
24												A																									1	1
25										A		A						B																			2	3
26			A																														B				1	2
28	A							B					A, X									B															2	4
29																																		A			1	1
30				B		B						B			B							B									A						1	5
31				A				B		B		B																						B			1	5
33			A				B								A	A								B	C		B						A				5	7
34			A					A				B			A													A						C			5	5
35										A		A																									2	2
36														A									B					A				A			A		4	5
37															A								A														2	2
38																											A										1	1
39																	A																				1	1
40	A					A								A					B		A			C				C							A		6	6
41	A		A																																		2	2
42													A, X						A																		2	2
43			A													B					A				C								B				2	3
44			A			B		B																				A						A			3	4
45																			A						B									A			2	3
46				A										B						A			B														2	4
47		A	B, X	A															B				A				A		A				A				6	8
48	A																	B									B, X					A					2	4
49	C		A																B																		1	1
51																													A								1	1
52			A					C		A					A	B, X																		A			4	5
53			B					A																													1	1
54															B, X	A														C							1	2
56				A																																	1	1
57																												B									0	0
58	A																	A		C				C		A											4	3
59	A		A			B					B	B		A							A																4	6
60				A																																	1	1
61			A					B																													1	2
62			A																A						C												2	2
63			A		A			A							A			A										C		C		A					8	6
64				A		A						B								A														C			3	4
65			A									B													C												1	2
66																A																					1	1
69	A																									A											2	2
71																											A										1	1
72									A												A												A				3	3
73	A								B				A, X	B		B, X						B						C									3	5
74				A								B																									1	2
76										B																											0	1
79										A				A	A																A						4	4
80																				A																	1	1
81				A		B		B		A																											2	3
82										C																											1	0
83														A								B												A			2	3
85				B										A									A														2	3
87			A										A, X																A		A					B	4	4
90								B																													0	0
91						A				B							A						A														3	4
92						A				A																						A					3	3
93								B															A									C					1	2
94	A											A			A																						3	3
95																				A			A														2	2
96	A			A		A		B					B		A				B		A		A				B						A				7	11
97			A																	B																	1	2
98										A																						A					2	2
99	A				A			B	B								A																	C	A		5	5
102		A																			A													A			3	3
103	A																																	A			2	2
104	A															A													A								3	3
C: Confirmed Luminex	0	0	0	0	0	0	NA	0	0	1	0	0	0	0	1	0	0	0	0	1	0	NA	0	0	1	0	0	4	0	1	0	0	0	2	0	NA	11	-
C: Non-confirmed Luminex	1	0	0	0	0	0	NA	1	0	0	0	0	0	0	0	0	0	0	0	0	0	NA	0	2	3	0	0	0	0	1	0	1	0	1	1	NA	11	-
B: Confirmed NGS	0	0	2	4	0	1	1	12	1	3	1	7	3	1	3	4	2	3	3	1	0	5	2	1	1	0	4	0	0	0	0	0	2	2	0	0	-	69
B: Non-confirmed NGS	0	0	1	0	2	4	0	2	1	0	0	0	0	1	0	0	0	0	1	0	0	0	0	0	0	0	0	1	0	0	0	1	0	0	0	1	-	15
A: Both methods	17	2	19	11	2	7	NA	3	1	8	0	6	4	8	8	5	3	2	5	4	7	NA	10	1	0	2	3	5	4	0	3	7	8	8	5	NA		178
Negative both methods	85	101	79	84	99	90	101	73	99	88	101	83	93	92	88	90	96	95	91	96	96	93	89	98	97	101	92	93	99	101	100	94	91	88	97	102		3355
% agreement	99.0	100	95.1	92.2	98.1	94.2	98.1	73.8	97.1	93.2	98.1	86.4	94.2	97.1	93.2	92.2	96.1	94.2	93.2	97.1	100	90.3	96.1	96.1	94.2	100	92.2	95.1	100	98.1	100	98.1	96.1	93.2	99.0	99.0		

HPV-genotype labels	
(grey box)	Not included in the Luminex assay
**HPV genotyping explanation**	
X	Mismatch(es) found in Luminex probe target by NGS
(orange-bordered box)	Closest match for genotype found by NGS
(pink box)	Exclusively Luminex positive sample row
(light blue box)	Exclusively NGS positive sample row
