# Supplementary figures and images for: HPV Genotyping of Modified General Primer-Amplicons Is More Analytically Sensitive and Specific by Sequencing than by Hybridization
Source: PLoS One. 2017 Jan 3;12(1):e0169074. doi: 10.1371/journal.pone.0169074 (PMC5207713; doi:10.1371/journal.pone.0169074)

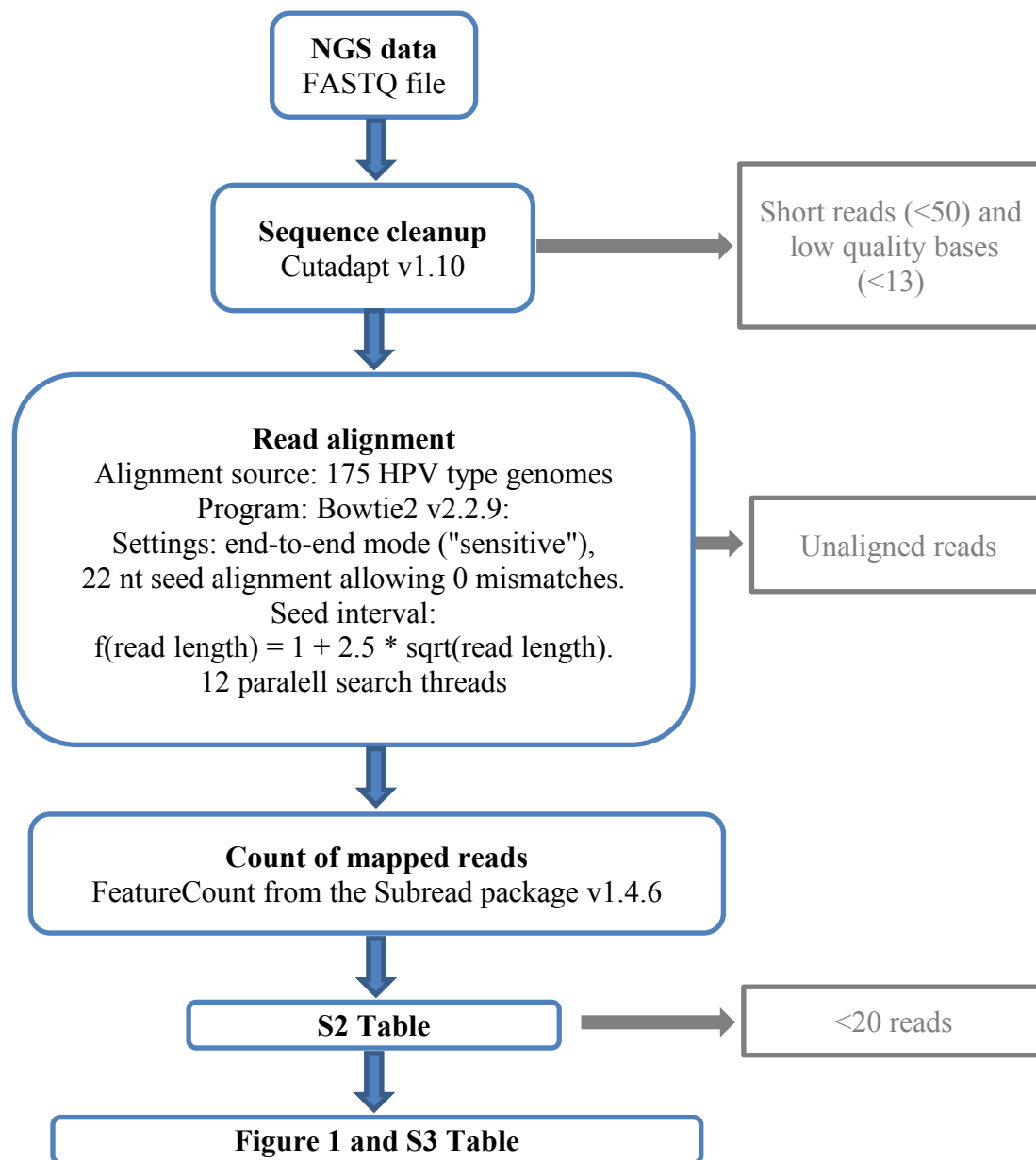

**S4 Table.** Schematic overview of the NGS bioinformatic pipeline and data analysis.

Supplement: S4 Table — (PDF) [file pone.0169074.s004.pdf]

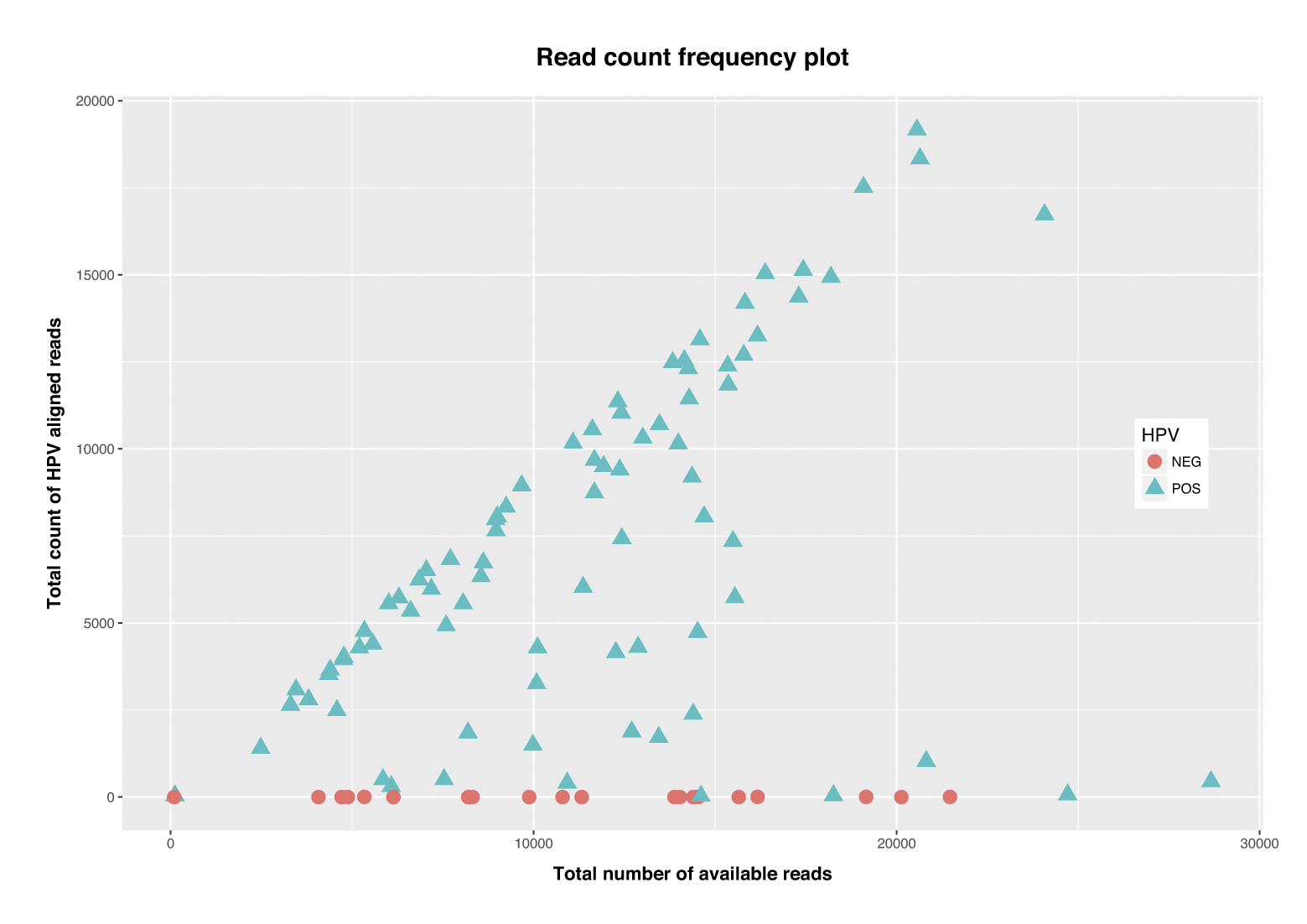

Supplement: S1 Fig — (TIF) [file pone.0169074.s005.tif]
