# Supplementary material for: HPV Genotyping of Modified General Primer-Amplicons Is More Analytically Sensitive and Specific by Sequencing than by Hybridization
Source: PLoS One. 2017 Jan 3;12(1):e0169074. doi: 10.1371/journal.pone.0169074 (PMC5207713; doi:10.1371/journal.pone.0169074)
Supplement: S2 Fig — (PDF) [file pone.0169074.s006.pdf]

S2 Figure: Flow chart of sample processing for hybridization and NGS assays

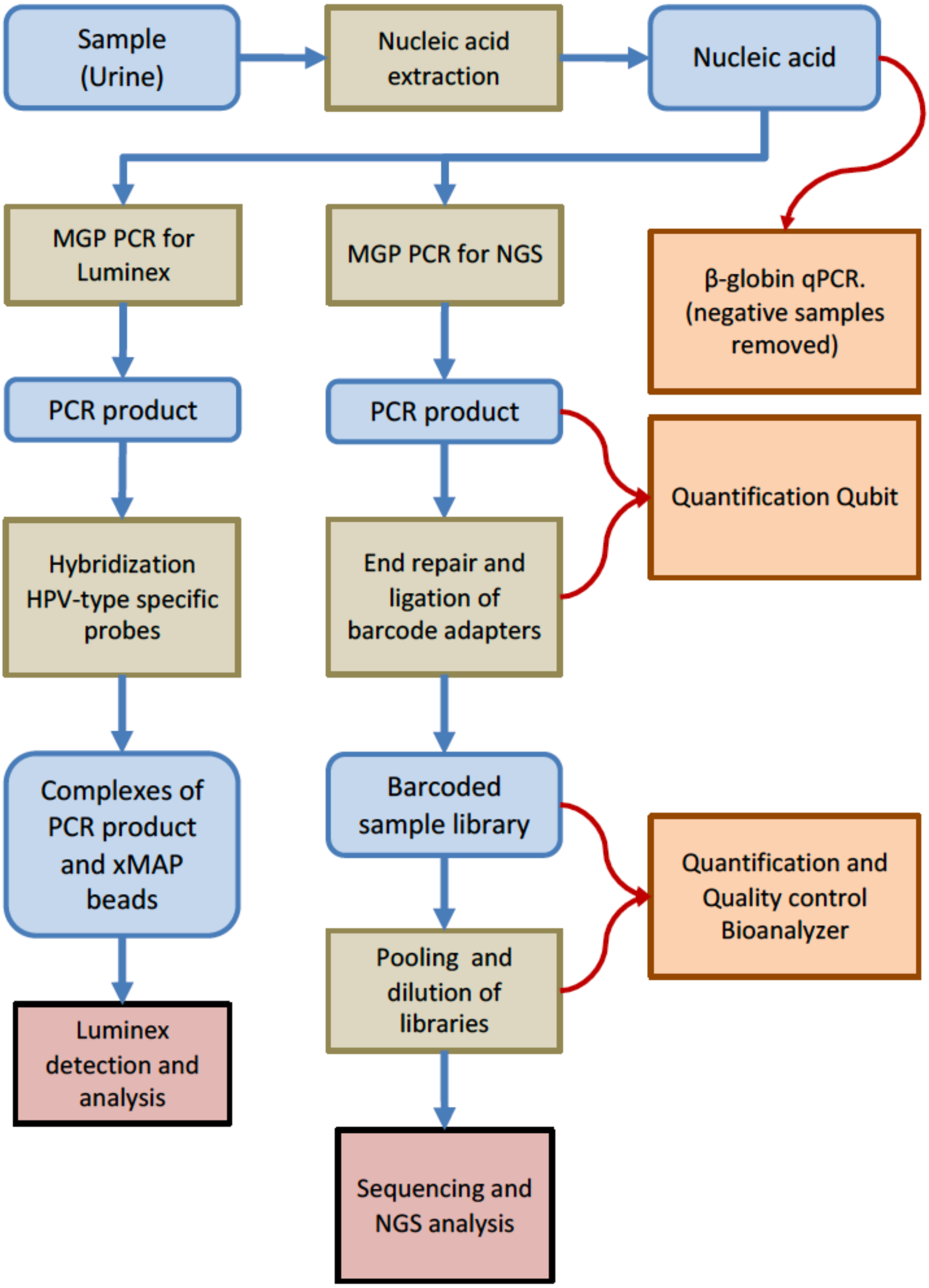

Sample processing for both methods, hybridization and NGS assays. Blue lines assign the flow of each individual sample. Blue boxes represent the sample characteristics at different stages of processing, green boxes represent the processes applied to the sample, orange boxes connected with red lines represent the quality and/or quantitation control performed at different stages during processing. The red boxes represent the final detection and data analyses of the two assays.
